# Nanog, in Cooperation with AP1, Increases the Expression of E6/E7 Oncogenes from HPV Types 16/18

**DOI:** 10.3390/v13081482

**Published:** 2021-07-28

**Authors:** Yakelin Díaz-Tejeda, Miriam C. Guido-Jiménez, Helga López-Carbajal, Alfredo Amador-Molina, Rocío Méndez-Martínez, Patricio Gariglio-Vidal, Marcela Lizano, Alejandro García-Carrancá

**Affiliations:** 1 Doctorado en Ciencias Biomédicas, Instituto de Investigaciones Biomédicas, Universidad Nacional Autónoma de México, Mexico City 04510, Mexico; ydiaztejeda@gmail.com; 2 Departamento de Biología Molecular y Biotecnología, Instituto de Investigaciones Biomédicas, Universidad Nacional Autónoma de México, Mexico City 04510, Mexico; mguido@iibiomedicas.unam.mx; 3 Subdirección de Investigación Básica, Instituto Nacional de Cancerología, Secretaría de Salud, Mexico City 14080, Mexico; phdamador@gmail.com (A.A.-M.); rosmen3244@gmail.com (R.M.-M.); 4 Departamento de Genética y Biología Molecular, Centro de Investigación y de Estudios Avanzados, Instituto Politécnico Nacional, Mexico City 07360, Mexico; vidal@cinvestav.mx; 5 Departamento de Medicina Genómica y Toxicología Ambiental, Instituto de Investigaciones Biomédicas, Universidad Nacional Autónoma de México, Mexico City 04510, Mexico; 6 Unidad de Investigación Biomédica en Cáncer, Instituto de Investigaciones Biomédicas, Universidad Nacional Autónoma de México & Instituto Nacional de Cancerología, Secretaría de Salud, Mexico City 14080, Mexico; satek1805@hotmail.com

**Keywords:** HPV16/18, LCR, Nanog, E6/E7, AP1, c-Jun

## Abstract

Persistent infections with some types of human papillomavirus (HPV) constitute the major etiological factor for cervical cancer development. Nanog, a stem cell transcription factor has been shown to increase during cancer progression. We wanted to determine whether Nanog could modulate transcription of E6 and E7 oncogenes. We used luciferase reporters under the regulation of the long control region (LCR) of HPV types 16 and 18 (HPV16/18) and performed RT-qPCR. We found that Nanog increases activity of both viral regulatory regions and elevates endogenous E6/E7 mRNA levels in cervical cancer-derived cells. We demonstrated by in vitro mutagenesis that changes at Nanog-binding sites found in the HPV18 LCR significantly inhibit transcriptional activation. Chromatin immunoprecipitation (ChIP) assays showed that Nanog binds in vivo to the HPV18 LCR, and its overexpression increases its binding as well as that of c-Jun. Surprisingly, we observed that mutation of AP1-binding sites also affect Nanog’s ability to activate transcription, suggesting cooperation between the two factors. We searched for putative Nanog-binding sites in the LCR of several HPVs and surprisingly found them only in those types associated with cancer development. Our study shows, for the first time, a role for Nanog in the regulation of E6/E7 transcription of HPV16/18.

## 1. Introduction

Persistent infections with certain types of human papillomavirus (HPV) belonging to the *Alphapapillomavirus* genus are considered necessary for the development of cervical cancer [1,2]. Although 16, 18, 31, 33, 35, 39, 45, 51, 52, 56, and 59 are known as high-risk (hr-HPV) types, most prominent members found in uterine cervix tumors are 16 and 18 [3].

HPV genomes are divided into three regions: a regulatory one encompassing the origin of replication and binding sequences for a plethora of transcription factors, called long control region (LCR), an early region containing non-structural genes (E1, E2, E4, E5, E6, and E7), and a late region containing L1 and L2 structural genes [4]. The LCR comprises roughly 800–900 bp and contains the early promoter driving expression of E6 and E7 oncogenes and a transcriptional enhancer with multiple transcription factor-binding sites (TBSs), including AP1, NF1, YY1, Sox2, and Oct1 [5,6,7].

Initiation and maintenance of tumors requires the continuous expression of E6 and E7 [8]. E7 and E6 viral oncoproteins promote immortalization and transformation of host cells through the degradation of cellular key regulatory proteins, including the product of the retinoblastoma gene (pRb) and p53. Additionally, cellular factors contribute to tumorigenicity in cooperation with HPV [9,10]. Nanog, which is an essential factor for stem cells, might be implicated.

Nanog is involved in carcinogenesis and has been associated with angiogenesis [11], epithelial–mesenchymal transition [12], multidrug resistance [13], worse overall survival, poor disease-free survival and lymph node metastasis [14], radio- [15] and immune-resistance [16], cancer progression and poor prognosis [17], and finally, invasion [18]. Nanog has also been proposed as a prognostic marker in various types of cancers, including non–small cell lung cancer [19], oral squamous cell carcinoma [20,21], ovarian tumors [22], urothelial carcinoma [23], glioblastoma [24,25], gastrointestinal cancer [26], colorectal cancer [27], and breast carcinoma [28]. In particular, both Nanog and hr-HPV E6 and E7 oncogenes have been associated with the progression of cervical cancer [29,30,31,32]. Importantly, it has been previously reported that E7 increases Nanog expression both in vitro and in vivo [33].

It has been shown that two AP1-binding sites are found in the LCR of HPV18 [34]. Furthermore, when AP1 is activated by a mutant Ras^V12^ protein, increased expression of E6 and E7 oncogenes is observed [35]. The principal component of the heterodimeric factor AP1 is c-Jun, which is involved in tumorigenesis, proliferation, invasion, migration, and angiogenesis [36]. c-Jun has also been implicated in the increase of E6/E7 oncogenes [37,38]. Since Nanog is known to bind to the c-Jun promoter and activate its expression, we hypothesized that Nanog could cooperate with AP1 to regulate E6 and E7 expression.

The aim of this work was to investigate whether Nanog affects transcriptional activity of the LCR from HPV16/18 and consequently expression of E6 and E7 oncogenes. We determine if Nanog binds in vivo to the LCR of HPV18. In addition, we wanted to know if putative Nanog-binding sites can be found in the LCRs of other HPVs and if their presence could be associated with cancer development.

## 2. Materials and Methods

### 2.1. Cell Culture

Human cervical cancer-derived cell lines HeLa and SiHa were authenticated by SRT DNA profiling at the University of Colorado DNA Sequencing and Analysis Core according to issued report number DP0297. The epithelial carcinoma-derived cell line C-33A was acquired from ATCC, Manassas, VA (#HTB-31^TM^; RRID:CVCL_1094). Human embryonic kidney cell line immortalized with SV40 T antigen (HEK-293T) was kindly provided by Dr. Manzo Merino from the National Cancer Institute in Mexico City (INCAN). Mouse epithelial primary tumor lung cell line TC-1 was generously donated by Dr. Lutz Gissmann from the German Cancer Research Center (DKFZ). Cells were cultured in Dulbecco’s Modified Eagle Medium (DMEM) containing 10% heat-inactivated fetal bovine serum, 50 U/mL penicillin and 50 μg/mL streptomycin from Gibco^®^, Waltham, MA (#12800017, #16000044, and #15140-122, respectively) and maintained at 37 °C in a humidified atmosphere containing 5% CO_2_.

### 2.2. Plasmids and Cloning

pGL3-basic, pcDNA 3.1 (empty vector or V), and pcDNA 3.1-Nanog (Nanog or N) coding for Nanog were obtained from Promega, (U47295), Invitrogen (#V79020), and Addgene (#28221; RRID:Addgene_28221), respectively. The vector expressing a mutant H-RasV12G (H-RasV12 gene or R) was previously described [39]. The LCR from HPV18 was cloned upstream of the luciferase reporter gene in pGL3 to generate pLCR18luc (LCR18). The LCR from HPV16 driving expression of the luciferase (LCR16) gene was previously described [40]. Plasmids containing mutations at two AP1-binding sites found in the HPV18 LCR directing luciferase gene expression were generated by subcloning preexisting mutated LCRs [41] into pGL3. The plasmid coding for B-galactosidase (Bgal) was obtained from Promega (E1081) and used for evaluating transfection efficiency. A plasmid coding for human c-Jun was previously described [42].

### 2.3. Site-Directed Mutagenesis

Mutations sites were generated using pLCR18luc as a template and the following primers: for site N: 5′-TATGTTGTATGTTACTATATTTG TTGGTTAT GTGGCGGGCAATAAAATATGTTTTGTGGTTCTGTGTGTTATGTG-3′, and for site N/A-E: 5′-CGCATATAGGCCCACCTGGTGCGAGTCATTTTCCTGTCCAGG-3′. We employed the QuikChange Lightning Multi Site-Directed Mutagenesis Kit (Agilent #210513) that includes a derivative of PfuUltra high-fidelity (HF) DNA polymerase. pLCR18luc was used together with a synthetic oligonucleotide primer that was extended during temperature cycling. Products were treated with Dpn I endonuclease to digest parental DNA template and transformed into XL10-Gold ultracompetent cells. Mutations were confirmed by sequencing selected clones using a 3500 xL Genetic Analyzer from Applied Biosystems^TM^.

### 2.4. Lentivirus Production and Transduction

HEK-293T cells (600,000) were seeded in 6-well plates and transfected with 2.5 μg second-generation plasmids obtained from Addgene; psPax2 for packaging, pMD2.G for envelope, pSin-EF2-Nanog-Pur for transference, or pL-SIN-EF1α-EGFP for transference control (#12260, RRID:Addgene_12260; #12259, RRID:Addgene_12259; #16578, RRID:Addgene_16578; and #21320, RRID:Addgene_21320, respectively). Lipofectamine 3000 (Invitrogen, Waltham, MA, USA, # L30000075) was used following the manufacture’s protocol, to improve lentiviral production. Transduction was performed on HeLa cells using one-half of the supernatant harvest obtained from 1 well of a 6-well plate for both Nanog and GFP produced lentiviruses. The transduction was made in the absence of serum and antibiotics and in the presence of the cationic polymer polybrene (Sigma-Aldrich, Burlington, MA, USA, # H9268) at a concentration of 10 μg/mL. After incubation for three hours at 37 °C, media were supplemented with 5% serum. On the third day, medium was changed, and cells were collected the same day or on day 7. Cells were seeded as follows: for 3-day collection, 750,000 cells on P100 plates; for 7-day collection, 350,000 on P100 plates.

### 2.5. Transfections

For luciferase assays, cells were transfected using PolyFect Transfection Reagent (Qiagen, Düsseldorf, GE, USA, #301105) in 6 well plates with 1 μg of LCR16/18 reporter vectors, together with 0.5 or 1μg of Nanog or H-RasV12 expression vectors, or 1 μg of empty vector. The following number of cells were used: TC-1, 250,000; C-33A, 290,000; and HEK-293T, 300,000. For RNA isolation, cells were transfected on 60 mm cell culture dishes with 2.5 μg of plasmids expressing Nanog or H-RasV12 using PolyFect reagent with the following number of cells: for HeLa, 450,000; for SiHa, 900,000. For western blots, reverse transfections were performed as follows: 10 μg of Nanog or H-RasV12 expressing vectors were mixed in 250 μL of DMEM medium with 32 μL of PolyFect and left 30 min at room temperature. Cells were seeded into 60 mm cell culture dishes and DNA-PolyFect mixture added. We used the following number of cells: TC-1, 1,500,000; C-33A, 2,000,000; HEK-293T 1,800,000; HeLa, 1,350,000; SiHa, 2,700,000.

### 2.6. Western Blot

Cells were treated with trypsin washed with PBS and lysed with RIPA buffer. Sixty micrograms of protein were loaded onto 12% polyacrylamide gels, transferred to 0.2 μm nitrocellulose membranes and blocked with 5% low-fat milk. Then, antibodies were added and incubated overnight. We used the following antibodies from Santa Cruz, Dallas, TX for Nanog (#sc-134218, RRID:AB_2150400), diluted 1/1000 in 5% low-fat milk; α-tubulin (#sc32293, RRID:AB_628412), diluted 1/20,000 in 5% low-fat milk; Anti Ras antibodies from Calbiochem, Burlington, MA (#OP40, RRID:AB_213400) were diluted 1/1000 in 0.2% albumin, and anti -mouse secondary antibody coupled to horseradish peroxidase from R&D, Minneapolis, (#HAF007, RRID:AB_357234) diluted 1/3000 in 5% low-fat milk. Chemo-luminescence was detected employing the Immobilon HRP substrate (Millipore, Burlington, MA, USA, #WBKLSO500) with a C-DiGiT (LI-COR, Biosciences, Lincoln, NB, USA) scanner.

### 2.7. Isolation of RNA, cDNA Synthesis and RTqPCR

RNA was extracted 48 h after transfection using Trizol (Invitrogen, Waltham, MA, USA, cat. number 15596018) as described in the manual (MAN0016385) and DNA digested with DNAase I (Invitrogen, Waltham, MA, USA, #18068015). The presence of the 28S and 18S was evaluated on 1% agarose gels. The RNA was quantified on a spectrophotometer, and a PCR was performed employing the GAPDH primers to ensure that no DNA was left. Then 1 μg of RNA per sample was used for retro-transcription using the RevertAid First Strand Kit (Thermo ScientificTM, Waltham, MA, USA, #K1691). cDNA was obtained and the qRT PCR was performed by using Thermo Maxima SYBR Green/ROX 1qPCR Master Mix (Thermo ScientificTM, Waltham, MA, USA, #K0251). The following primers were used: E6-18-F: 5′-GCG ACC CTA CAA GCT ACC TG-3′, E6-18-R: 5′-GTT GGA GTC GTT CCT GTC GT-3′; E7-18-F: 5′-AAC ATT TAC CAG CCC GAC GA-3′, E7-18-R: 5′-TCG TCT GCT GAG CTT TCT AC-3′; E6-16-F: 5′-ACT GCA ATG TTT CAG GAC CC-3′, E6-16-R: 5′-TCA GGA CAC AGT GGC TTT TG-3′; E7-16-F: 5′-CCC AGC TGT AAT CAT GCA TG-3′, E7-16-R: 5′-TGC CCA TTA ACA GGT CTT CC-3′; GAPDH-F: 5′-AAG GTC GGA GTC AAC GGA TTT-3′, GAPDH-R: 5′-CCA TGG GTG GAA TCA TAT TGG AA-3′. PCR cycles were as follows: 95 °C/10 min, 35 cycles: 95 °C/35 s; 60 °C/ 35 s; 72 °C/35 s. Data were processed with the Delta CT method.

### 2.8. Luciferase Assays

Cells were washed 48 h after transfection, treated with trypsin, and then suspended in 30 μL of lysis buffer, followed by five cycles of freezing on dry ice and heating at 37 °C for 45 s, homogenizing the mix using a vortex after every heating step. The Dual Luciferase Reporter Assay kit was used (Promega, Madison, WI, USA, #E1910), and both the luciferase and the β-galactosidase activities were detected in a GloMax 20/20 Luminometer (Promega). For β-galactosidase, a buffer containing 100 mM sodium phosphate pH 8, 1 mM magnesium chloride, and Galacton 1X (Thermo Scientific^TM^, Waltham, MA, USA, #T2120) was used. Additionally, a reaction accelerator compound (Thermo Scientific Waltham, MA, USA, #T2222) was employed at the moment of detection, and the signal was measured after 5 s.

### 2.9. ChIP Assays

Chromatin immunoprecipitation was performed using the One Day ChIP Kit (Diagenode, Denville, NJ, USA, #C01010080). Three 100 mm dishes with HeLa cells at 85% confluence were employed per condition. Cells were fixed in 1% formaldehyde, quenched with 125 mM glycine and lysed with a buffer containing 1% SDS, 10 mM EDTA (pH 8.0), 50 mM Tris-HCL (pH 8.0), and 1X protease inhibitors (Roche #04693159001). Chromatin was fragmented with an Ultrasonic Processor (Gene Q, model GEX500 SOVC505-00), using 1 cycle of 37% amplitude for 30 s, alternating 5 s ON/3 s OFF. The size of the fragments was determined to be between (200–500 bp). Protein was quantified by using the Pierce BCA Protein Assay Kit (ThermoFisher #23225). For each immunoprecipitation, 700 μg of protein was used with the following antibodies: anti-Nanog (Cell Signaling, Danvers, MA, USA, #5232S, RRID:AB_10561315), anti c-Jun (Abcam #ab31419, RRID:AB_731605), and anti IgG (Diagenode, Denville, NJ, USA, kch-504-250).

Quantitative PCR was performed as described previously. As negative control, primers for a region on chromosome 7 were used for both Nanog and c-Jun immunoprecipitations: F: 5′-TCTAGGCTAGGGTAGTGAAGCAC-3′ and R: 5′-GGGACAGCCAGTGATGAAGAAA-3′. As positive control for Nanog, the following primers from a segment of the Nanog promoter were used: F: 5′-GTCTGGGTTACTCTGCAGCTACT-3′ and R: 5′-CTTAGACC CACCCCTCCTGGC-3′. For c-Jun positive control, the following primers for the cyclin D promoter were used: F: 5′-CCAGGGCAAATTCTA AAGGT-3′ and R: 5′-CACACCTCTGAATGGAAAGC-3′. Finally, for HPV18 LCR, the following primers were used: F: 5′-CTTTTGGGCACTGCTCCTAC-3′ and R: 5′-GCAGTTTTATTACTTAGGGAGTGGA-3′. PCR was performed as described above, except that for annealing, temperatures for primers described were as follows: 60 °C for c-Jun and Nanog negative control; 62 °C for Nanog positive control, 58 °C for c-Jun positive control, and 59 °C for the LCR.

The efficiency of each primer was calculated based on the results obtained from a standard curve made from different input dilutions. The occupancy of c-Jun and Nanog was calculated based on the negative control IgG. The formulas employed were extracted from the Diagenode Kit protocol (C01010055).

### 2.10. In Silico Sequence Analysis

Putative Nanog-binding sites as reported by Mitsui et al. 2003 (C(G/C)ATTAN) [43], Lin et al. 2015 (GCATTAC) [44], or Das et al. 2012 (GAATTAC) [45] were searched in LCR sequences from 12 different HPVs (5, 6, 8, 11, 16, 18, 31, 35, 45, 51, 52, and 56) and 2 PV that infect macaques (MmPV1 & MmPV2). Sequences were analyzed using Multiple Sequence Alignment-Clustal W2 (RRID:SCR_002909). LCR sequences from different papillomavirus genomes were obtained from the NCBI nucleotide site (Supplementary Table S1).

### 2.11. Statistical Analysis

Statistical analysis was performed by one-way ANOVA and Tukey’s multiple comparison tests (GraphPad Prism Software, Inc. RRID:SCR_002798). *p*-Values under 0.05 were considered significant. Three replicates were considered for each experiment.

## 3. Results

### 3.1. Nanog Increases Transcriptional Activity from the LCR of HPV Types 16 and 18

**Figure 1:**
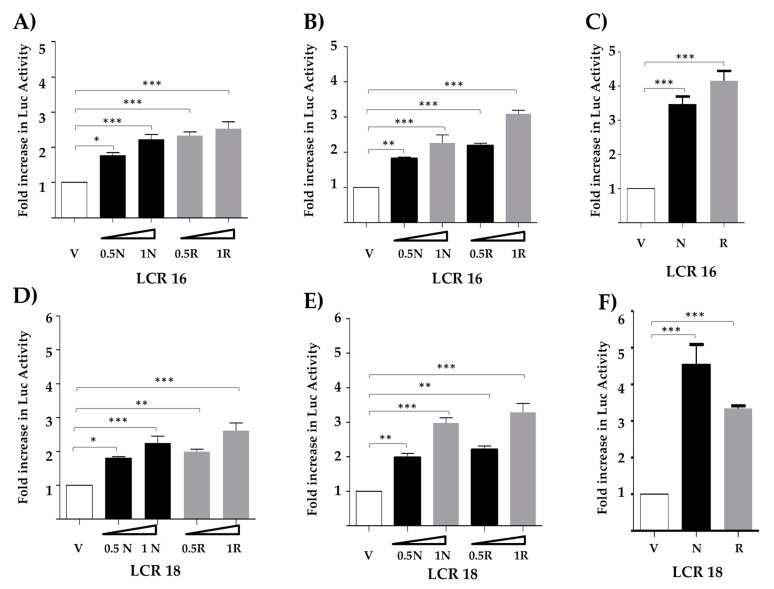
Nanog increases the transcriptional activity of HPV16/18 LCRs in different transformed cell lines. Three different transformed cells lines were used: TC-1 (**A**,**D**); C-33A (**B**,**E**); and HEK-293T (**C**,**F**) were transfected with reporter vectors containing the HPV16 LCR (LCR16) or HPV18 LCR (LCR18), respectively, driving the expression of the luciferase gene, and co-transfected with empty vector (V); Nanog (N); or H-Ras^V12^ (R), as a positive control. Different amounts of N and R vectors (0.5 μg and 1 μg) were used in the case of TC-1 and C-33A cells. Luciferase reporter activity was measured 48 h post-transfection. The standard error of the mean of three independent experiments, in triplicate, is depicted in the graph. One-way ANOVA test with a Tukey analysis was performed to evaluate the significant differences. * *p* ≤ 0.05, ** *p* ≤ 0.01, *** *p* ≤ 0.001.

In order to determine if Nanog could modulate transcription of E6 and E7, we first co-transfected vectors containing the LCR of either HPV type 16 or 18 cloned upstream of the luciferase gene, together with empty vector or 0.5 and 1 μg of plasmid expressing Nanog. We evaluated changes of luciferase activity in three different transformed cell lines (TC-1, C-33A, and HEK-293T) and found that exogenous Nanog significantly increased reporter activity in all of them, as shown in Figure 1. In TC-1 and C-33A cells we observed a dose–response increment of luciferase activity driven by the LCR of HPV type 16 (Figure 1A,B) or 18 (Figure 1D,E). A significant difference in reporter activity was observed also in HEK-293T cells when 1 ug of the plasmid expressing Nanog was co-transfected (Figure 1C,F). As expected, co-transfections with a plasmid expressing mutated H-Ras (Ras^V12^) as positive control also showed a significant increase in reporter activity. Expression of Nanog and H-Ras^V12^ was verified by Western blot in reverse transfected cells (Figure S1). We then conclude that Nanog positively regulates transcriptional activity from both HPV 16 and 18 regulatory regions.

### 3.2. The Expression of E6 and E7 Is Increased by Exogenous Nanog in Both HPV16/18-Positive Cancer-Derived Cell Lines

**Figure 2:**
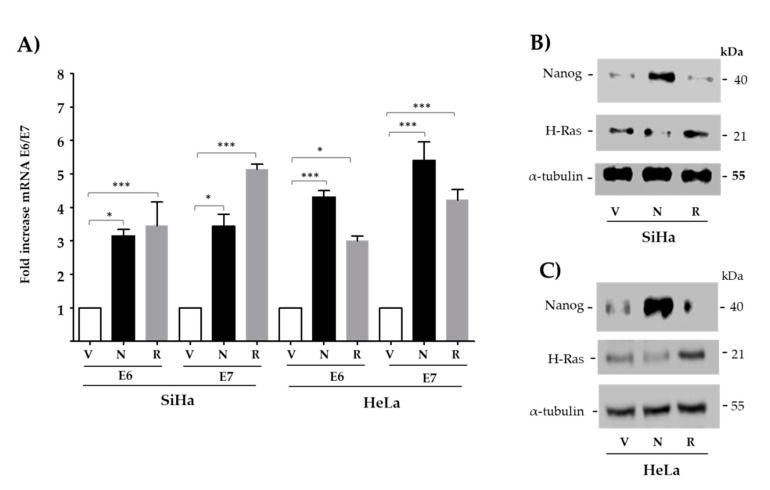
Nanog increases E6 and E7 expression. (**A**) SiHa and HeLa cells were transiently transfected with empty vector (V), Nanog (N), and H-Ras^V12^ (R), as a positive control. (**A**) RT-qPCR of E6 and E7 was performed. The means and ± SD of three independent experiments (in triplicate) are depicted in the graph. One-way ANOVA test with a Tukey analysis was performed to evaluate the significant differences. * *p* < 0.05, *** *p* ≤ 0.001. (**B**,**C**) SiHa and HeLa cells were reverse transfected and Nanog or H-RasV12 protein expression evaluated using α-tubulin as protein loading control.

The effect of transfected Nanog on endogenous E6 and E7 oncogene expression was evaluated by RT-qPCR in SiHa and HeLa cell lines, positive for types 16 and 18, respectively. Both cervical cancer-derived cell lines exhibited a significant increase in the levels of E6 and E7 when transfected with Nanog. A 3-fold to 5-fold significant increment in expression of E6 and E7 was observed in comparison with empty vector (Figure 2A). When only transfection reagent was used as negative control, similar results were obtained (Figure S2). As expected, H-Ras^V12^ significantly increased viral oncogene expression to similar levels. Higher levels of Nanog and H-Ras^V12^ proteins in transfected cells was corroborated, as shown in Figure 2B,C.

### 3.3. Nanog- and AP1-Binding Sites Are Necessary for LCR-Mediated Activation Along 

**Figure 3:**
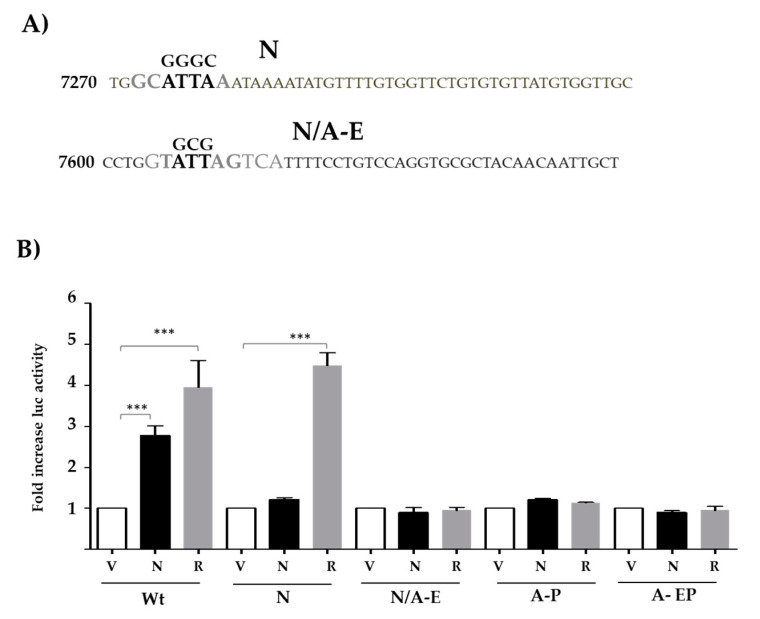
Mutations in Nanog- and AP1-putative-binding sites preclude the increase in HPV 18 LCR transcriptional activity mediated by Nanog. (**A**) Representation of Nanog sites on the HPV18 LCR (N & N/A-E) and mutations introduced (above). (**B**) Luciferase activity was evaluated in HEK-293T cells co-transfected with reporter vectors containing either wild type (Wt), mutations at Nanog site (N), Nanog-AP1 shared site (N/A-E), or single (A-P) or double mutants (A-EP) of AP1 sites, together with empty vector (V), Nanog (N), or H-Ras^V12^ (R) as a positive control. Luciferase activity was measured 48 h after transfection. The standard error of the mean of three independent experiments, in triplicate, is depicted. One-way ANOVA test with Tukey analysis was performed to evaluate the significant differences. *** *p* ≤ 0.001.

To test the relevance and function of putative Nanog-binding sites found in the LCR of HPV18, we performed site-directed mutagenesis. Reporter plasmids containing the LCR were used to create independent mutations of either the N or N/A-E sites, as indicated in Figure 3A (substituted nucleotides are indicated above core elements shown in bold). The N/A-E site includes binding elements for both Nanog and AP1. Plasmids containing either wild-type sequences or single-site mutations were co-transfected along with Nanog, negative, or positive controls. Overexpression of Nanog showed no effect on reporter activity when either N or N/A-E sites were mutated (Figure 3B). Surprisingly, when either the proximal AP1-binding (A-P) site or both sites (A-EP) are mutated, no increase in reporter activity was observed in the presence of Nanog. Therefore, intact Nanog and AP1-binding are necessary to increase transcriptional activity of the HPV18 LCR.

### 3.4. Nanog Binds In Vivo to the HPV18 LCR and Cooperates with C-Jun to Increase Transcriptional Activity

**Figure 4:**
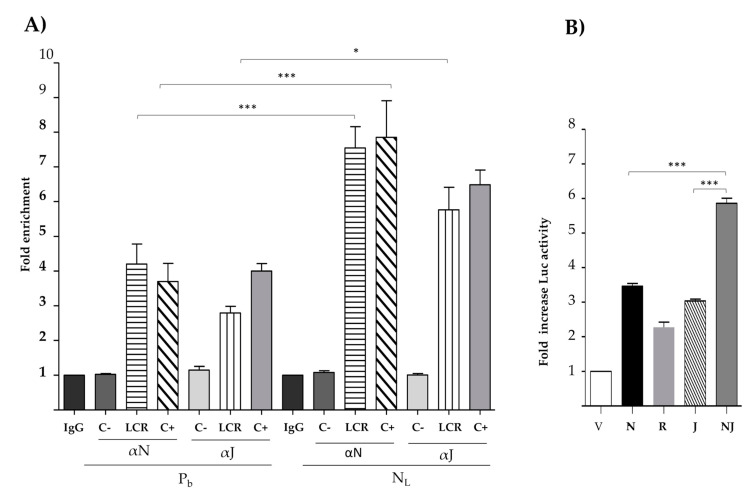
Nanog and c-Jun cooperate to increase LCR activity. (**A**) Chromatin immunoprecipitation assay (ChIP) was performed on HeLa cells treated with vehicle (P_b_), left, or cells transduced with lentivirus expressing Nanog and collected seven days after (N_L_), right. Chromatin was immunoprecipitated with anti Nanog (αN) and anti c-Jun (αJ) antibodies. Anti IgG antibody was used as isotype negative control (IgG). qRT-PCR was performed with primers for the HPV18 LCR to measure Nanog and c-Jun binding (LCR) and a region of chromosome 7, as a negative control (C-) for both Nanog and c-Jun. Nanog promoter and cyclin D1 promoter (C+) were used as positive controls for Nanog and c-Jun, respectively. (**B**) TC-1 cells were transiently transfected with vectors containing the HPV18 LCR (LCR 18), driving the expression of the luciferase gene, and co-transfected with empty vector (V), Nanog (N), H-Ras^V12^ (R), and c-Jun (J) alone or in combination with N (NJ). The luciferase reporter activity was measured 48 h post-transfection. The standard error of the mean of three independent experiments, in triplicate, is depicted in the graph. One-way ANOVA test with a Tukey analysis was performed to assess the significant differences. * *p* <0.05, *** *p* ≤ 0.001.

We first corroborated that Nanog increases the expression of c-Jun, as previously described [44]. HeLa cells were transfected with V, N, or R. c-Jun protein levels were measured and, as expected, c-Jun expression increased in cells transfected with Nanog (Figure S3). In order to determine whether Nanog binds in vivo to the HPV18 LCR and promotes c-Jun binding to this region, we conducted a chromatin immunoprecipitation assay (Figure 4A). HeLa cells treated only with polybrene (P_b_) agent as negative control showed 4.2-fold and 2.8 enrichment of both Nanog and c-Jun, respectively. In contrast, cells transduced with lentivirus coding for Nanog (N_L_) displayed 7.5- and 5.8-fold enrichment for Nanog and c-Jun, respectively. We thus conclude that Nanog is capable of binding to the HPV18 LCR in vivo and, when overexpressed, increases its binding to the LCR together with c-Jun.

To determine whether Nanog and c-Jun together are able to increase the activity of HPV18 LCR more than N or J separately, we performed a luciferase assay. TC-1 cells were transfected with the HPV18 LCR and co-transfected with V, N, R, J, or N and J together (NJ). We observed a 5.9-fold increase in luciferase activity in the condition where NJ was co-transfected, compared with N alone (3.5-fold) or J alone (3.0-fold) (Figure 4B). Therefore, the combined effect of Nanog and c-Jun is stronger than either of these factors alone.

### 3.5. Identification of Nanog-Binding Sites in LCRs of Different Alpha- and Betapapillomaviruses Associated with Cancer Development

In order to evaluate if Nanog-binding sites shown here to activate transcription of E6/E7 are found in the regulatory regions of other viral types, we analyze LCR sequences from several HPVs. In addition, we performed a comparative analysis of the putative-binding sites in HPV associated or not with cancer development. We performed an in-silico analysis of LCR sequences from different HPV types, searching for putative Nanog-binding sites that could contain elements related to sequences previously described (43, 45).

We searched LCR sequences from HPV types belonging either to the *Betapapillomavirus* genus associated with skin cancer (5 and 8) or to the *Alphapapillomavirus* genus associated with either low-grade lesions (6 and 11) or high-grade lesions and cancer (16, 18, 31, 35, 45, 51, 52, and 56). Surprisingly, our analysis clearly shows that while the LCR of HPV types associated with cancer contain at least one putative Nanog-binding site, no such sites were found in the LCR of both HPV types 6 and 11, not associated with cancer. The majority of putative Nanog-binding sites were found positioned at equivalent distances to the E6/E7 transcription start site. In addition, we also found an equivalent site in the LCR of MmPV2. Sequences corresponding to putative Nanog-binding found in the LCR of different papillomavirus genomes are shown in Table 1.

Our results support the notion that Nanog increases expression of E6/E7 from HPV types 16 and 18 by activating transcription from the LCR. In addition, we showed that Nanog binds in vivo and activates transcription from the LCR of HPV18 in cooperation with AP-1. Importantly, we only found putative Nanog-binding sites in the LCRs of HPV types belonging to both the alpha and beta genus that have been associated with cancer development.

## 4. Discussion

Expression of E7/E6 has been shown to be regulated by several transcription factors associated with cancer, pluripotency, and regulation of stem cells. Nanog has been shown to be expressed in malignant tumors of the uterine cervix, and high expression associated with poor tumor prognosis [46,47].

In this work, we studied the effect of Nanog in the transcriptional regulation of E6/E7 from HPV types 16 and 18. In order to find out if Nanog affects viral oncogene expression, we first performed reporter gene assays using the respective LCRs (Figure 1) and three different transformed cells lines. We observed that Nanog significantly increases transcriptional activities from the both LCRs. To our knowledge, this is the first report indicating a role of Nanog in the regulation of E6/E7 from hr-HPVs. Importantly, it has been reported that KLF-4, another cancer stem factor, binds the HPV31 promoter and increases viral transcription through two regions of the HPV31 LCR, activating both early and late promoters [48]. Furthermore, binding of Sox2 to the HPV16 LCR [42], and FOXA1 and MYC to HPV16/18 promoter and regulatory sequences has been reported [49].

Next, we demonstrated that ectopically expressed Nanog significantly enhances transcription of E6 and E7 oncogenes from HPV16/18 (Figure 2) in two tumor-derived cell lines containing integrated copies of viral DNA and expressing E6/E7. It is interesting to note that increased expression of Nanog has been shown in cancer stem cells from uterine cervix-derived cells [50].

To determine whether Nanog binds directly to the HPV18 LCR, we mutated two putative Nanog-binding sites (Figure 3B), in accordance with reported data [43,44,45]. Surprisingly, we observed that when either of these Nanog-binding sites was mutated, transcriptional activity was abolished.

Alternatively, we tested an HPV18 LCR mutated at both AP1-binding sites to explore the participation of AP1 in the increase in LCR transcriptional activity mediated by Nanog. Contrary to what happened with wt HPV18 LCR, Nanog was unable to activate LCR with mutated AP1 sites, indicating that transcriptional activation of the LCR mediated by Nanog requires AP1 binding. It has been reported that Nanog is upregulated by c-Jun and that c-Jun is positively regulated by Nanog [44,51] The c-Jun oncogene binds with high affinity to DNA as a heterodimer, forming the AP1 factor, where c-Jun can bind to any member of the c-Jun, c-Fos, ATF/cyclic AMP-responsive-element-binding (CREB), and Maf families [52]. Interestingly, it has been demonstrated that AP1 increases the activity of the hr-HPV LCRs, as well as the expression of E6 and E7 oncogenes [53,54].

Moreover, to determine if Nanog can bind to the HPV LCRs, we performed chromatin immunoprecipitation assays. We showed that Nanog binds to the LCR of HPV18 in vivo, and its overexpression increases the levels of c-Jun bound to the LCR (Figure 4A). This result could be explained by the fact that normally Nanog binds its target DNA as a complex with other factors. In fact, Nanog and c-Jun have been reported to bind to each other in vivo [44]. Therefore, it is not surprising that Nanog and AP1 can interact and modulate transcriptional activity of the LCR. As expected, we showed that Nanog and c-Jun cooperatively increased transcriptional activity more than either of these factors alone (Figure 4B).

A role of Nanog in transcriptional regulation of E6/E7 in transformed cells is supported by the fact that at least one DNA sequence similar to those shown here is present in the LCR of 10 different HPV types associated with cancer. In contrast, no similar sites are found in the LCR of HPV types 6 and 11, implicating a possible role of Nanog in the transformation process. In addition, a sequence related to site NE was found in the LCR of MmPV2. It is important to note that in types 18, 35, and 45, Nanog and AP1 sites (GTATTAGTCA) are found overlapped and in conserved positions. In the case of MmPV1, where no Nanog-putative-binding sites could be documented, we noticed the presence of two putative AP1-binding sites that could cooperate to activate E6 and E7 transcription. Since all our experiments were performed using transformed cells, it could be important to determine if similar effects are observed in cell lines that maintain episomal copies of the viral genome.

Our results contribute to the understanding of the relationship that stem cell transcription factors could have on the transcriptional activation of E6 and E7 from hr-HPV, during the transformation process. Thus, it is possible that infection of stem cells with hr-HPV can potentiates transformation. 

## Figures and Tables

**Table 1:** Nanog putative binding sites found in the LCR of *Alpha- and Betapapillomavirus*.

Genus	Species	Type	Position	Nanog-Putative- Binding Sites	Bases To +1
*Alpha*	9	16	7171–7177	5′- tatgttGAATTAGtgttgt-3´	823
		31	7813–7819	5′- tgtcatGCATTATaaataa-3´	199
			7861–7867	5′- atatagGTATTACaccgtt-3´	152
		35	7565–7771	5′- cttgcaGTATTAGtcattt-3´	386
			7761–7767	5′- ctcactGTATTACacattg-3´	193
		52	7334–7340	5′- gtgtatGTATTAAtaaagta-3´	705
	5	51	7241–7247	5′- atgtggGTATTACattatc-3´	703
	6	56	7617–7623	5′- caccctGTATTACtcacag-3´	322
	7	18	7272–7278	5′- tatgtgGCATTAAataaaa-3´	683
			7605–7611	5′- cacctgGTATTAGtcattt-3´	350
		45	7603–7609	5′- cacctgGTATTAGtcattt-3´	350
	12	MmPV2	7206–7212	5′- gttaaaGTATTAAtaaagg-3´	664
*Beta*	1	5	7669–7675	5′- atgctcGGATTAGggacct-3´	268
		8	7576–7582	5′- atgctcGGATTAGgtcgcc-3´	267

## Supplementary Materials

The following are available online at https://www.mdpi.com/article/10.3390/v13081482/s1, Figure S1: Western blot of (A) TC-1, (B) C-33A, and (C) HEK-293T transfected cells, Figure S2: Nanog increases E6 and E7 expression, Figure S3: Nanog increases the expression of c-Jun protein, Figure S4: Western blot of Nanog and Jun in HeLa and TC-1 cells, Table S1: HPV sequences analyzed.

## References

[1] Walboomers J.M., Jacobs M.V., Manos M.M., Bosch F.X., Kummer J.A., Shah K.V., Snijders P.J., Peto J., Meijer C.J., Munoz N. (1999). Human papillomavirus is a necessary cause of invasive cervical cancer worldwide. J. Pathol..

[2] zur Hausen H. (2002). Papillomaviruses and cancer: From basic studies to clinical application. Nat. Rev. Cancer.

[3] Bosch F.X., Burchell A.N., Schiffman M., Giuliano A.R., de Sanjose S., Bruni L., Tortolero-Luna G., Kjaer S.K., Munoz N. (2008). Epidemiology and natural history of human papillomavirus infections and type-specific implications in cervical neoplasia. Vaccine.

[4] Burley M., Roberts S., Parish J.L. (2020). Epigenetic Regulation of Human Papillomavirus Transcription in the Productive Virus Life Cycle. Semin. Immunopathol..

[5] O’Connor M., Bernard H.U. (1995). Oct-1 Activates the Epithelial-Specific Enhancer of Human Papillomavirus Type 16 via a Synergistic Interaction with NFI at a Conserved Composite Regulatory Element. Virology.

[6] Bernard H.U. (2013). Regulatory elements in the viral genome. Virology.

[7] Harden M.E., Munger K. (2017). Human papillomavirus molecular biology. Mutat. Res. Rev. Mutat. Res..

[8] Prati B., Marangoni B., Boccardo E. (2018). Human Papillomavirus and Genome Instability: From Productive Infection to Cancer. Clinics.

[9] Chesters P.M., McCance D.J. (1989). Human papillomavirus types 6 and 16 in cooperation with Ha-ras transform secondary rat embryo fibroblasts. J. Gen. Virol..

[10] Chong T., Chan W.K., Bernard H.U. (1990). Transcriptional activation of human papillomavirus 16 by nuclear factor I, AP1, steroid receptors and a possibly novel transcription factor, PVF: A model for the composition of genital papillomavirus enhancers. Nucleic Acids Res..

[11] Kohler E.E., Cowan C.E., Chatterjee I., Malik A.B., Wary K.K. (2011). NANOG induction of fetal liver kinase-1 (FLK1) transcription regulates endothelial cell proliferation and angiogenesis. Blood.

[12] Qin S., Li Y., Cao X., Du J., Huang X. (2017). NANOG regulates epithelial-mesenchymal transition and chemoresistance in ovarian cancer. Biosci. Rep..

[13] Song K.H., Choi C.H., Lee H.J., Oh S.J., Woo S.R., Hong S.O., Noh K.H., Cho H., Chung E.J., Kim J.H. (2017). Upregulation by NANOG Promotes Multidrug Resistance and a Stem-like Phenotype in Immune Edited Tumor Cells. Cancer Res..

[14] Zhao L., Liu J., Chen S., Fang C., Zhang X., Luo Z. (2018). Prognostic significance of NANOG expression in solid tumors: A meta-analysis. OncoTargets Ther..

[15] Dehghan Harati M., Rodemann H.P., Toulany M. (2019). Nanog Signaling Mediates Radioresistance in ALDH-Positive Breast Cancer Cells. Int. J. Mol. Sci..

[16] Kim S., Cho H., Hong S.O., Oh S.J., Lee H.J., Cho E., Woo S.R., Song J.S., Chung J.Y., Son S.W. (2020). LC3B upregulation by NANOG promotes immune resistance and stem-like property through hyperactivation of EGFR signaling in immune-refractory tumor cells. Autophagy.

[17] Basati G., Mohammadpour H., Emami Razavi A. (2020). Association of High Expression Levels of SOX2, NANOG, and OCT4 in Gastric Cancer Tumor Tissues with Progression and Poor Prognosis. J. Gastrointest. Cancer.

[18] Kashyap T., Nath N., Mishra P., Jha A., Nagini S., Mishra R. (2020). Pluripotency Transcription Factor Nanog and Its Association with Overall Oral Squamous Cell Carcinoma Progression, Cisplatin-Resistance, Invasion and Stemness Acquisition. Head Neck.

[19] Chang B., Park M.J., Choi S.I., In K.H., Kim C.H., Lee S.H. (2017). NANOG as an adverse predictive marker in advanced non–small cell lung cancer treated with platinum-based chemotherapy. OncoTargets Ther..

[20] Kim H.M., Kang Y.H., Byun J.H., Jang S.J., Rho G.J., Lee J.S., Park B.W. (2017). Midkine and NANOG Have Similar Immunohistochemical Expression Patterns and Contribute Equally to an Adverse Prognosis of Oral Squamous Cell Carcinoma. Int. J. Mol. Sci..

[21] Rodrigo J.P., Villaronga M.A., Menéndez S.T., Hermida-Prado F., Quer M., Vilaseca I., Allonca E., Mallo D.P., Astudillo A., García-Pedrero J.M. (2017). A Novel Role for Nanog As An Early Cancer Risk Marker in Patients with Laryngeal Precancerous Lesions. Sci. Rep..

[22] Kenda Šuster N., Frković Grazio S., Virant-Klun I., Verdenik I., Smrkolj Š. (2017). Cancer Stem Cell-Related Marker NANOG Expression in Ovarian Serous Tumors: A Clinicopathological Study of 159 Cases. Int. J. Gynecol. Cancer.

[23] Md Akhir M.K.A., Hussin H., Veerakumarasivam A., Choy C.S., Abdullah M.A., Abd Ghani F. (2017). Immunohistochemical expression of NANOG in urothelial carcinoma of the bladder. Malays. J. Pathol..

[24] Soni P., Qayoom S., Husain N., Kumar P., Chandra A., Ojha B.K., Gupta R.K. (2017). CD24 and Nanog expression in Stem Cells in Glioblastoma: Correlation with Response to Chemoradiation and Overall Survival. Asian Pac. J. Cancer Prev..

[25] Kuciak M., Mas C., Borges I., Sánchez-Gómez P., Ruiz A. (2019). Chimeric NANOG repressors inhibit glioblastoma growth in vivo in a context-dependent manner. Sci. Rep..

[26] Liang C., Zhao T., Ge H., Xu Y., Ren S., Yue C., Li G., Wu J. (2018). The clinicopathological and prognostic value of Nanog in human gastrointestinal luminal cancer: A meta-analysis. Int. J. Surg..

[27] Wang H., Liu B., Wang J., Li J., Gong Y., Li S., Wang C., Cui B., Xue X., Yang M. (2017). Reduction of NANOG Mediates the Inhibitory Effect of Aspirin on Tumor Growth and Stemness in Colorectal Cancer. Cell Physiol. Biochem..

[28] Emadian Saravi O., Naghshvar F., Torabizadeh Z., Sheidaei S. (2019). Immunohistochemical Expression of Nanog and Its Relation with Clinicopathologic Characteristics in Breast Ductal Carcinoma. Iran. Biomed. J..

[29] Ye F., Zhou C., Cheng Q., Shen J., Chen H. (2008). Stem-cell-abundant proteins Nanog, Nucleostemin and Musashi1 are highly expressed in malignant cervical epithelial cells. BMC Cancer.

[30] Gu T.-T., Liu S.-Y., Zheng P.-S. (2012). Cytoplasmic NANOG-positive stromal cells promote human cervical cancer progression. Am. J. Pathol..

[31] Liu S., Minaguchi T., Lachkar B., Zhang S., Xu C., Tenjimbayashi Y., Shikama A., Tasaka N., Akiyama A., Sakurai M. (2018). Separate analysis of human papillomavirus E6 and E7 messenger RNAs to predict cervical neoplasia progression. PLoS ONE.

[32] Chopra S., Deodhar K., Pai V., Pant S., Rathod N., Goda J.S., Sudhalkar N., Pandey P., Waghmare S., Engineer R. (2019). CD44, and Outcomes Following Chemoradiation in Locally Advanced Cervical Cancer: Results From a Prospective Study. Int. J. Radiat. Oncol. Biol. Phys..

[33] Organista-Nava J., Gómez-Gómez Y., Ocadiz-Delgado R., García-Villa E., Bonilla-Delgado J., Lagunas-Martínez A., Tapia J.S., Lambert P.F., García-Carrancá A., Gariglio P. (2016). The HPV16 E7 oncoprotein increases the expression of Oct3/4 and stemness-related genes and augments cell self-renewal. Virology.

[34] García-Carrancá A., Thierry F., Yaniv M. (1988). Interplay of viral and cellular proteins along the long control region of human papillomavirus type 18. J. Virol..

[35] Medina-Martínez O., Vallejo V., Guido M.C., García-Carrancá A. (1997). Ha-ras oncogene–induced transcription of human papillomavirus type 18 E6 and E7 oncogenes. Mol. Carcinog..

[36] Priyadarshini R., Hussain M., Attri P., Kaur E., Tripathi V., Priya S., Dhapola P., Saha D., Madhavan V., Chowdhury S. (2018). BLM Potentiates c-Jun Degradation and Alters Its Function as an Oncogenic Transcription Factor. Cell Rep..

[37] Muñoz J.P., Carrillo-Beltrán D., Aedo-Aguilera V., Calaf G.M., León O., Maldonado E., Tapia J.C., Boccardo E., Ozbun M.A., Aguayo F. (2018). Tobacco Exposure Enhances Human Papillomavirus 16 Oncogene Expression via EGFR/PI3K/Akt/c-Jun Signaling Pathway in Cervical Cancer Cells. Front Microbiol..

[38] Morgan E.L., Scarth J.A., Patterson M.R., Christopher W., Georgia W.C., Barba-Moreno H.D., Macdonald A. (2020). E6-mediated activation of JNK drives EGFR signalling to promote proliferation and viral oncoprotein expression in cervical cancer. Cell Death Differ..

[39] Miranda E.I., Santana C., Rojas E., Hernández S., Ostrosky-Wegman P., García-Carrancá A. (1997). Induced mitotic death of HeLa cells by abnormal expression of c-H-ras. Mutat. Res. Mol. Mech. Mutagen..

[40] Martínez-Ramírez I., Del-Castillo-Falconi V., Mitre-Aguilar I.B., Amador-Molina A., Carrillo-García A., Langley E., Zentella-Dehesa A., Soto-Reyes E., García-Carrancá A., Herrera L.A. (2017). SOX2 as a New Regulator of HPV16 Transcription. Viruses.

[41] Thierry F., Spyrou G., Yaniv M., Howley P. (1992). Two AP1 Sites Binding JunB Are Essential for Human Papillomavirus Type 18 Transcription in Keratinocytes. J. Virol..

[42] Hirai S.I., Bourachot B., Yaniv M. (1990). Both Jun and Fos contribute to transcription activation by the heterodimer. Oncogene.

[43] Mitsui K., Tokuzawa Y., Itoh H., Segawa K., Murakami M., Takahashi K., Maruyama M., Maeda M., Yamanaka S. (2003). The homeoprotein Nanog is required for maintenance of pluripotency in mouse epiblast and ES cells. Cell.

[44] Lin Y., Xiong F., Zhou Y., Wu X., Liu F., Xue S., Chen H. (2015). NANOG upregulates c-Jun oncogene expression through binding the c-Jun promoter. Mol. Carcinog..

[45] Das S., Jena S., Kim E.M., Zavazava N., Levasseur D.N. (2012). Transcriptional Regulation of Human NANOG by Alternate Promoters in Embryonic Stem Cells. J. Stem Cell Res. Ther..

[46] Ruiz G., Valencia-González H.A., Pérez-Montiel D., Muñoz F., Ocadiz-Delgado R., Fernández-Retana J., Pérez-Plasencia C., Reséndis-Antonio O., Gariglio P., García-Carrancá A. (2019). Genes Involved in the Transcriptional Regulation of Pluripotency Are Expressed in Malignant Tumors of the Uterine Cervix and Can Induce Tumorigenic Capacity in a Nontumorigenic Cell Line. Stem Cells Int..

[47] Han G.H., Cho H. (2020). 203 High expression of Nanog and CRY1 is involved with tumor progression and poor prognosis in patients with cervical cancer. Int. J. Gynecol. Cancer.

[48] Gunasekharan V.K., Li Y., Andrade J., Laimins L.A. (2016). Post-Transcriptional Regulation of KLF4 by High-Risk Human Papillomaviruses Is Necessary for the Differentiation-Dependent Viral Life Cycle. PLoS Pathog..

[49] Sichero L., Sobrinho J.S., Villa L.L. (2012). Identification of Novel Cellular Transcription Factors that Regulate Early Promoters of Human Papillomavirus Types 18 and 16. J. Infect. Dis..

[50] Ortiz-Sánchez E., Santiago-López L., Cruz-Domínguez V.B., Toledo-Guzmán M.E. (2016). Hernández-Cueto, D.; Muñiz-Hernández, S.; Garrido, E.; Cantú De León, D.; García-Carrancá. A. Characterization of cervical cancer stem cell-like cells: Phenotyping, stemness, and human papilloma virus co-receptor expression. OncoTarget.

[51] Ibrahim E.E., Babaei-Jadidi R., Saadeddin A., Spencer-Dene B., Hossaini S., Abuzinadah M., Li N., Fadhil W., Ilyas M., Bonnet D. (2012). Embryonic NANOG Activity Defines Colorectal Cancer Stem Cells and Modulates through AP1- and TCF-Dependent Mechanisms. Stem Cells.

[52] Garces de los Fayos Alonso I., Liang H.C., Turner S.D., Lagger S., Merkel O., Kenner L. (2018). The Role of Activator Protein-1 (AP-1) Family Members in CD30-Positive Lymphomas. Cancers.

[53] Liu Y., Li J.Z., Yuan X.H., Adler-Storthz K., Che Z. (2002). An AP-1 Binding Site Mutation in HPV-16 LCR Enhances E6/E7 Promoter Activity in Human Oral Epithelial Cells. Virus Genes.

[54] Kyo S., Klumpp D.J., Inoue M., Kanaya T., Laimins L.A. (1997). Expression of AP1 during cellular differentiation determines human papillomavirus E6/E7 expression in stratified epithelial cells. J. Gen. Virol..

