# Supplementary material for: Nanog, in Cooperation with AP1, Increases the Expression of E6/E7 Oncogenes from HPV Types 16/18
Source: Viruses. 2021 Jul 28;13(8):1482. doi: 10.3390/v13081482 (PMC8402821; doi:10.3390/v13081482)
Supplement: Supplementary file 1 [file viruses-13-01482-s001.zip › viruses-1242541-supplementary.pdf]

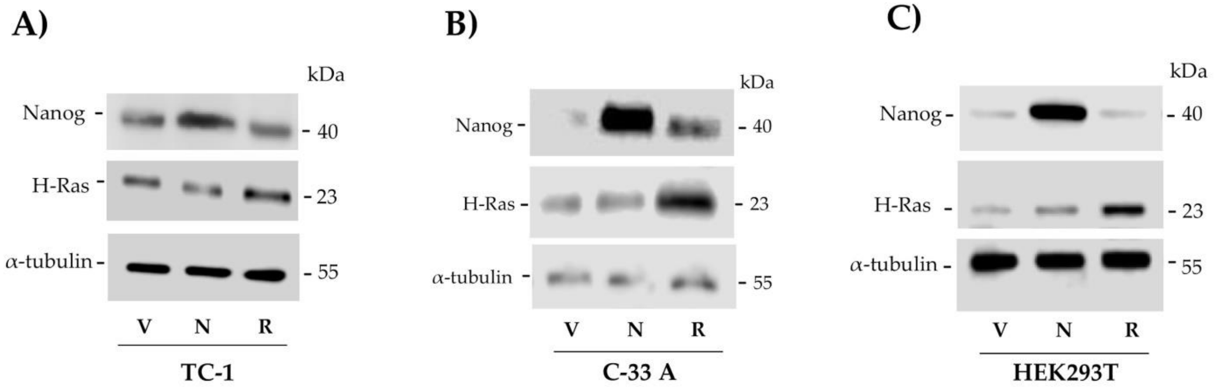

Figure S1. Western Blot of **A**) TC-1, **B)** C-33A and **C)** HEK-293T transfected cells. N and R overexpression was measured; α-tubulin was used as protein loading control.

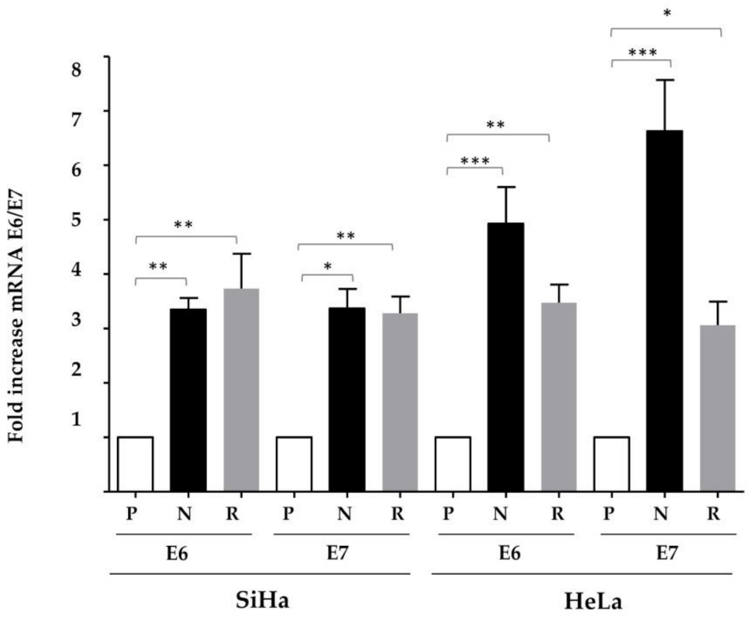

Figure S2. Nanog increases E6 and E7 expression. SiHa and HeLa cells were transiently transfected with transfectant agent (P); Nanog (N); and H-RasV12 (R), as a positive control. qRT-PCR of E6 and E7 was performed. Standard Error of the Mean of three independent experiments is shown. One way ANOVA test with a Tukey analysis was performed to evaluate the significant differences. *$p < 0.05$,**$p \leq 0.01$, ***$p \leq 0.001$.

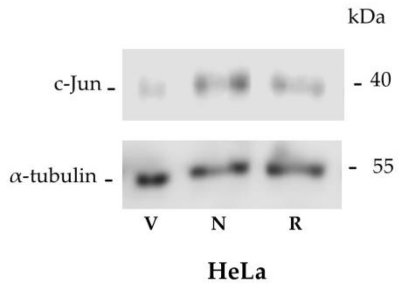

Figure S3. Nanog increases the expression of protein c-Jun. HeLa cells were transiently transfected with empty vector(V), Nanog (N) and H-RasV12 as a positive control. α-tubulin was used as protein loading control

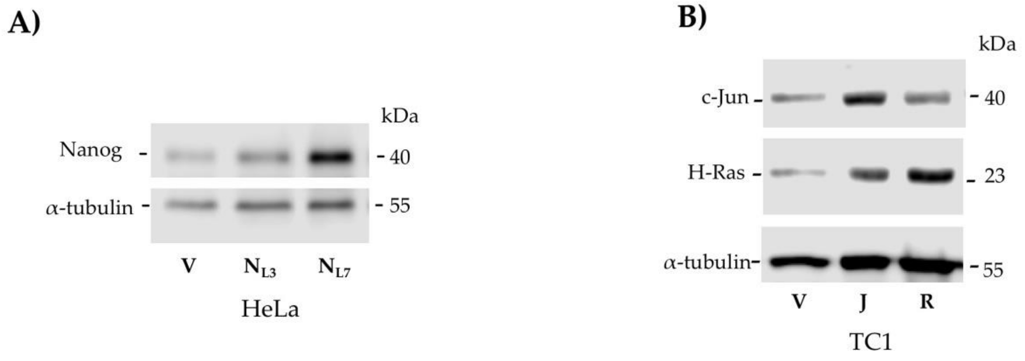

Figure S4. Western Blot of Nanog and Jun in HeLa and TC1 cells. A) Nanog lentivirus expression at day 3 (NL3) and day 7 (NL7) of transduction in HeLa cells, compared with polybrene (Pb) treated cells. B) c-Jun vector expression on HeLa cells. α-tubulin was used as protein loading control.

Table S1. HPV sequences analyzed.

GENUS	SPECIE	TYPE	LCR	GENBANK	REFERENCE
Alpha	10	**6**	7292/101	**NC_001355.1**	Schwarz E, Dürst M, Demankowski C, Lattermann O, Zech R, Wolfsperger E, Suhai S, zur Hausen H. DNA sequence and genome organization of genital human papillomavirus type 6b. EMBO J. 1983;2(12):2341-8. PMCID: PMC555455. PMID: 6321162
		**11**	7277/101	**M14119.1**	Dartmann K, Schwarz E, Gissmann L, zur Hausen H. The nucleotide sequence and genome organization of human papilloma virus type 11. Virology. 1986 May;151(1):124-30. doi: 10.1016/0042-6822(86)90110-8. PMID: 3008427
	9	**16**	7157/97	**K02718.1**	Seedorf K, Krämmer G, Dürst M, Suhai S, Röwekamp WG. Human papillomavirus type 16 DNA sequence. Virology. 1985 Aug;145(1):181-5. doi: 10.1016/0042-6822(85)90214-4. PMID: 2990099
		**31**	7067/107	**J04353.1**	Goldsborough MD, DiSilvestre D, Temple GF, Lorincz AT. Nucleotide sequence of human papillomavirus type 31: a cervical neoplasia-associated virus. Virology. 1989 Jul;171(1):306-11. doi: 10.1016/0042-6822(89)90545-x. PMID: 2545036
		**35**	7110/109	**M74117.1**	Lorincz AT, Quinn AP, Lancaster WD, Temple GF. A new type of papillomavirus associated with cancer of the uterine cervix. Virology. 1987 Jul;159(1):187-90. doi: 10.1016/0042-6822(87)90366-7. PMID: 3037779
		**52**	7155/101	**X74481.1**	Delius H, Hofmann B. Primer-directed sequencing of human papillomavirus types. Curr Top Microbiol Immunol. 1994; 186:13-31. doi: 10.1007/978-3-642-78487-3_2. PMID: 8205838.
	5	**51**	7036/96	**M62877.1**	Lungu O, Crum CP, Silverstein S. Biologic properties and nucleotide sequence analysis of human papillomavirus type 51. J Virol. 1991 Aug;65(8):4216-25. doi: 10.1128/JVI.65.8.4216-4225.1991. PMID: 1649326; PMCID: PMC248858.
	6	**56**	7098/101	**X74483.1**	Delius H, Hofmann B. Primer-directed sequencing of human papillomavirus types. Curr Top Microbiol Immunol. 1994;186:13-31. doi: 10.1007/978-3-642-78487-3_2. PMID: 8205838.
	7	**18**	7137/104	**NC_001357**	Cole ST, Danos O. Nucleotide sequence and comparative analysis of the human papillomavirus type 18 genome. Phylogeny of papillomaviruses and repeated structure of the E6 and E7 gene products. J Mol Biol. 1987 Feb 20;193(4):599-608. doi: 10.1016/0022-2836(87)90343-3. PMID: 3039146

		**45**	7150/101	**X74479.1**	Delius H, Hofmann B. Primer-directed sequencing of human papillomavirus types. Curr Top Microbiol Immunol. 1994; 186:13-31. doi: 10.1007/978-3-642-78487-3_2. PMID: 8205838.
	**12**	**MmPV1**	7468/199	**M60184.1**	Ostrow RS, LaBresh KV, Faras AJ. Characterization of the complete RhPV 1 genomic sequence and an integration locus from a metastatic tumor. Virology. 1991 Mar;181(1):424-9. doi: 10.1016/0042-6822(91)90519-h. PMID: 1847267.
		**MmPV2**	7061/7876	**MG837557.1**	Long T, Wong PY, Ho WCS, Burk RD, Chan PKS, Chen Z. Complete Genome Sequences of Six Novel Macaca mulatta Papillomavirus Types Isolated from Genital Sites of Rhesus Monkeys in Hong Kong SAR, China. Microbiol Resour Announc. 2018 Dec 6;7(22):e01414-18. doi: 10.1128/MRA.01414-18. PMID: 30533857
Beta	**1**	**5**	7468/199	**NC_001531.1**	Zachow KR, Ostrow RS, Faras AJ. Nucleotide sequence and genome organization of human papillomavirus type 5. Virology. 1987 May;158(1):251-4. doi: 10.1016/0042-6822(87)90263-7. PMID: 3033892.
		**8**	7396/195	**M12737.1**	Fuchs PG, Iftner T, Weninger J, Pfister H. Epidermodysplasia verruciformis-associated human papillomavirus 8: genomic sequence and comparative analysis. J Virol. 1986 May;58(2):626-34. doi: 10.1128/JVI.58.2.626-634.1986. PMID: 3009874
